# Extracellular Vesicles—Connecting Kingdoms

**DOI:** 10.3390/ijms20225695

**Published:** 2019-11-14

**Authors:** Eric Woith, Gregor Fuhrmann, Matthias F. Melzig

**Affiliations:** 1 Institute of Pharmacy, Pharmaceutical Biology, Dahlem Center of Plant Sciences, Freie Universität Berlin, Königin-Luise-Str. 2+4, D-14195 Berlin, Germany; melzig@zedat.fu-berlin.de; 2 Helmholtz Centre for Infection Research (HZI), Biogenic Nanotherapeutics Group (BION), Helmholtz Institute for Pharmaceutical Research Saarland (HIPS), Campus E8.1, 66123 Saarbrücken, Germany; 3 Department of Pharmacy, Saarland University, Campus E8.1, 66123 Saarbrücken, Germany

**Keywords:** extracellular vesicles, prokaryota, eukaryota, archaea, interkingdom communication, cross-kingdom RNAi

## Abstract

It is known that extracellular vesicles (EVs) are shed from cells of almost every type of cell or organism, showing their ubiquity in all empires of life. EVs are defined as naturally released particles from cells, delimited by a lipid bilayer, and cannot replicate. These nano- to micrometer scaled spheres shuttle a set of bioactive molecules. EVs are of great interest as vehicles for drug targeting and in fundamental biological research, but in vitro culture of animal cells usually achieves only small yields. The exploration of other biological kingdoms promises comprehensive knowledge on EVs broadening the opportunities for basic understanding and therapeutic use. Thus, plants might be sustainable biofactories producing nontoxic and highly specific nanovectors, whereas bacterial and fungal EVs are promising vaccines for the prevention of infectious diseases. Importantly, EVs from different eukaryotic and prokaryotic kingdoms are involved in many processes including host-pathogen interactions, spreading of resistances, and plant diseases. More extensive knowledge of inter-species and interkingdom regulation could provide advantages for preventing and treating pests and pathogens. In this review, we present a comprehensive overview of EVs derived from eukaryota and prokaryota and we discuss how better understanding of their intercommunication role provides opportunities for both fundamental and applied biology.

## 1. Introduction

**Figure 1:**
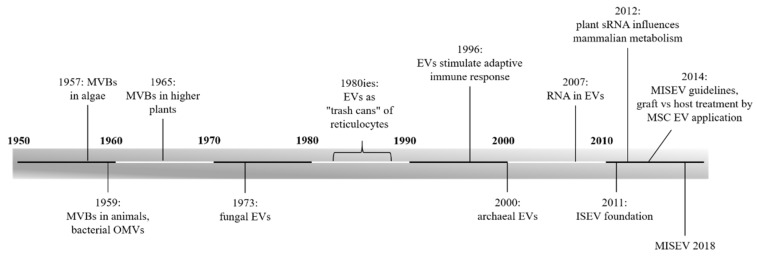
Timeline of extracellular vesicle (EV) research throughout all empires. First recognized, in the late 1950s, EVs have been found throughout all empires of life. Although they were initially underestimated as “trash cans”, diverse functions, as well as clinical applications, have been identified, such as their role as RNA transporters or their clinical use in regenerative medicine. ISEV, International Society for Extracellular Vesicles; MISEV, minimal information for studies of extracellular vesicles; MSC, mesenchymal stem cell; MVB, multi vesicular body; OMV, outer membrane vesicle; and sRNA, small noncoding RNA.

Currently, it is well-known that extracellular vesicles (EVs) play a role in diverse cellular communication processes. Initially, when they were discovered, no function could be identified. As shown in the timeline (Figure 1), the first observation of multivesicular bodies (MVBs), and thus of (intraluminal) exosomes which are the smallest class among EVs, occurred in the 1950s. MVBs were first recognized in algae [1] and mammalian cells [2]. At the same time, outer membrane vesicles (OMVs) were found in bacteria [3,4]. Nearly ten years after the detection in algae, in 1965, MVBs were found in higher plants [5]. Again, after almost a decade, in 1973, EVs were reported in fungi [6]. At that time, probably none of the researchers recognized the significance of the discovered structures. About two decades later, by investigating reticulocytes, exosomes were thought to facilitate cells getting rid of garbage. It was assumed that they simply “defenestrate” remnants, instead of degradation [7,8,9,10]. This assumption of waste disposal probably resulted from the juvenile state of research on EVs at that time. The year 1996 marked a turning point in the thinking of EVs, when Raposo et al. assumed EVs influenced antigen presentation in vivo [11]. Since then, EVs were no longer thought to function only as “trash cans”.

The ubiquity of EVs in all empires of life was confirmed in 2000, when they were accounted for in archaea [12]. In recent years, much knowledge on EVs has been gathered. Loaded with bioactive proteins, lipids, and nucleic acids, EVs enable long distance communication between cells. Information can either be transmitted by EV uptake (membrane fusion, endocytosis) into the recipient cell or via receptor interaction on the cell surface [13,14]. Because EVs shuttle nucleic acids, they facilitate post-transcriptional regulation of the recipient cell metabolism. It was revealed, in 2007, that EVs carry messenger RNA (mRNA) and small noncoding RNA (sRNA), enabling cells to exchange genetic information, and therefore the thinking of intercellular communication was renewed [15,16,17,18]. The key regulators are sRNAs, a class of single stranded RNA, comprising around 22 nucleotides. This sRNA binds complimentarily to mRNA, resulting in post-transcriptional gene silencing [19,20,21]. EVs protect this fragile cargo from degradation by RNases and appear to be responsible for targeting recipient cells [20,22,23,24,25,26,27,28]. Compared to cell-cell communication, utilized by low-molecular messenger substances (e.g., hormones), EVs further provide vehicles transmitting cargo in high concentrations, unaffected by diffusion or dilution [29].

EVs were observed throughout all empires of life, underlining their high evolutionary importance, however, to date, the major question about EVs remains unanswered, i.e., "What is their elementary function?” Although we cannot solve this conundrum, in this review, we have compiled information on EVs from different origins and vesicular cross talk between individuals, species, and even kingdoms.

## 2. Eukaryotic EVs

### 2.1. Animal EVs

EVs have been isolated from almost all types of mammalian cells or body fluids. Three main groups are widely accepted apoptotic bodies, microvesicles, and exosomes [30,31]. Apoptotic bodies (1000 to 5000 nm in diameter) result from cell fragmentation and budding. They occur as a product of programmed cell death to be phagocytosed. Microvesicles (100 to 1000 nm), also called microparticles, are shed directly by plasma membrane, while exosomes (30 to 150 nm) are released by fusion of MVBs with the plasma membrane [10,27,28,32,33]. Specific markers of EV subtypes are not yet universally accepted. Thus, the International Society for Extracellular Vesicles (ISEV) suggests the use of innocuous terms, referring to physical or biochemical characteristics, isolation conditions, or cell origin such as small EVs, tetraspanin CD9+ EVs, or macrophage derived EVs. The minimal information for studies of extracellular vesicles (MISEV) guidelines, published in 2014 and 2018, further define requirements for EV isolation, characterization, and quantification which researchers should respect when working with EVs, and therefore provide useful evaluations and recommendations of techniques and devices [34,35]. Analytic marker proteins, both localized on the membrane surface and in the vesicular matrix, are well described [34,35,36], especially the transmembrane tetraspanins CD9, CD63, and CD81 which are often used as markers, as well as for EV isolation and purification [32,37,38,39,40]. These and other markers are widely, but not generally accepted [35]. More extensive information regarding, especially, human EVs has been compiled in a couple of comprehensive reviews [13,16,31,33,41,42].

#### 2.1.1. EVs in Health and Diseases

EVs can apparently influence health. This is particularly reflected when physiological processes turn pathological. Disorders and stress can alter EV secretion, as neurodegenerative diseases such as Alzheimer’s and Parkinson´s disease, multiple sclerosis, amyotrophic lateral sclerosis, and strokes can cause changes to EV release. It remains to be clarified whether EVs have detrimental or protective effects on the progression of neurodegenerative diseases [43,44,45]. Although our knowledge of how EVs are involved into the pathology of certain diseases needs to be expanded, we can utilize them as biomarkers. For instance, several cancer types can be detected, even in early stages [45]. Apparently, EVs play a crucial role concerning infectious diseases. They elicit a bidirectional arms race in host–pathogen interaction (see also Section 4 Interindividual, Interspecies, and Inter-Kingdom Regulation). In order to stimulate the immune response, the infected cells send EVs, loaded with pathogen-associated molecular patterns (PAMPs) [46]. The list of human diseases, influenced by EVs, is constantly growing. Autoimmune disorders [47] are affected, as well as diseases of affluence, for example, atherosclerosis, obesity, and metabolic syndrome. Obesity and atherosclerosis have been shown to be associated with elevated EV numbers and altered composition [14]. In obese patients, adipocyte-derived exosomes could increase oxidative stress and progression of chronic inflammation [48].

**Figure 2:**
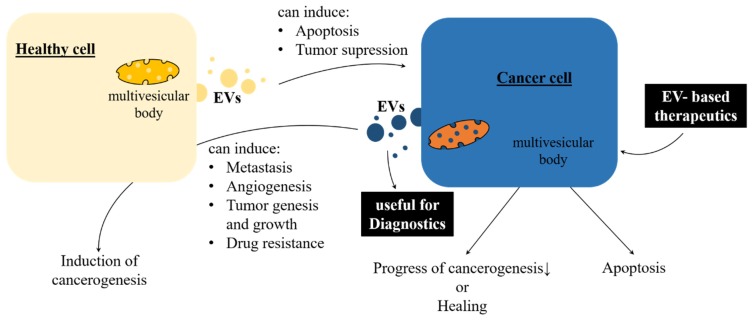
Bidirectional regulation of cancer via extracellular vesicles (EVs). Vesicular regulation can come to pass in both directions. Cancer cells can be affected positively by EVs from healthy cells, leading to tumor apoptosis or at least inhibition of progress. Meanwhile, EVs derived from cancer cells can also stimulate cancerogenesis or metastasis either in the microenvironment of the originating cell or in distant tissues. Additionally, EVs can be used as diagnostic tools, as well as therapeutic vehicles.

Of particular interest is the EV linkage between human health and diseases in the field of cancer treatment. On the one hand, EVs derived from dendritic cells have been shown to suppress murine tumor growth [49]. On the other hand, exosomes are involved in multiple therapeutically difficult cancer processes, such as tumor growth, tumorigenesis, angiogenesis, drug resistance, and metastasis [50,51,52]. These controversial facts lead to the conclusion that exosomal cell-to-cell communication in cancer is a bidirectional signaling pathway (Figure 2). One major topic in the current research on mammalian exosomes is whether they can be used to advance cancer therapy [53,54]. Nevertheless, EVs are promising biomarkers for distinct medicinal diagnostic purposes such as early and precise detection of several cancer types, infectious diseases, diabetes, autoimmune disorders, and neurodegenerative diseases [14,45,50,55,56,57].

#### 2.1.2. Therapeutic Applications of Mammalian EVs

In the field of EV-based therapeutics there are a few successful preclinical concepts that underline the potential of these natural nanoparticles. There are two main areas of interest for EV-therapeutics which are:

- their use in regenerative medicine;
- EVs as carriers for drugs.

In several studies, EVs from mesenchymal stem cells (MSCs) have been shown to have an inherent anti-inflammatory and immunomodulatory capacity [58] and may positively influence tissue regeneration after cardiac injury [59]. Since this finding, many preclinical assessments have used MSC EVs for repair of liver [60] and myocardial tissue [61]. An important example of the anti-inflammatory and immunomodulatory potential of MSC EVs was reported, in 2014, by Kordelas et al. [62]. They showed that life-threatening immune overreaction due to the graft-versus-host phenomenon could be ameliorated by sequential application of MSC EVs. Among the different potential therapeutic applications of EVs, the use of anti-inflammatory MSC EVs appears to be most advanced and it is important to find suitable regulatory guidelines for their production under good manufacturing practice conditions [45,63].

Especially in cancer therapy, nanoscale drug encapsulation is a quickly developing field. One major advantage of such approaches is that doses can be reduced due to increased bioavailability, leading to diminished toxicity of cytostatic agents. The number of semi- and synthetic nano-formulations is high, while only a few EV preparations are being investigated clinically [64].

Regarding the use of EVs as drug carriers, there appear to be a few important challenges to overcome when these approaches are to be tested preclinically. These challenges include EV-heterogeneity and characterization of EV population used and their reproducible production and loading with compounds of choice. Various loading methods are described in the literature, including passive incubation, saponin-assisted encapsulation, electroporation, ultrasound, and extrusion [65,66]. The efficiency of each of these methods appears to be highly dependent on the cellular source of EV and the physicochemical properties of encapsulated drugs. Moreover, specific pharmacokinetic properties of EVs, such as circulation kinetics and biodistribution profile [67], are not as understood as needed. For a detailed overview on these topics, the reader is referred to recent review manuscripts [65,68]. These manuscripts underline that the field of EV-therapeutics is already on the right track, but additional effort is needed to better clarify which physiological behavior EVs have when used as therapeutics, and which biological barriers they encounter when administered systemically [69].

Interestingly, the US Food and Drug Administration has recently approved the first RNA interference-based drug. As the active compound, siRNA is carried by lipid nanoparticles and intended to treat hereditary transthyretin amyloidosis. The nanoparticles prevent RNA degradation and facilitate entry into cells, which is exactly the same role that EVs are thought to have, however, these nanoparticles have limitations since they can show dose-limiting toxicity and their target is nearly exclusively the liver [68]. Liposomal formulations have been investigated since the 1960s, and thus they are probably the best investigated group in nanomedicine. Although modification can improve liposome properties, such difficulties have not yet been overcome satisfyingly [70,71,72]. Furthermore, it is noteworthy that in the tremendous list of organic nanomaterials, compiled by Palazzolo et al., there is only one single preparation that is applied orally, i.e., grape exosome-like nanoparticles loaded with curcumin [64]. Apparently, EVs (also from others than the animal kingdom) have underestimated properties.

Presently, sufficient amounts of EV production is a limiting aspect for therapeutic usage. Depending on the cultured cell type, yield can vary significantly. For example, 785 µg EVs have been isolated per million B16BL6 cells while one million MKN45 cells delivered 375 µg EVs after 72 h incubation, indicating that EV release is cell-type and incubation-time dependent [73,74]. Different approaches could be adopted to enhance the outcome, such as:

- stimulation of EV production [73,75,76];
- using bioreactors which can increase EV yields more than 100-fold as compared to conventional cell cultures [74];
- exploration of alternative EV sources.

Alternative sources for EV production can predictively be either animals (e.g., bovine milk [77]) or plants. Fungal and prokaryotic EVs appear to be inappropriate due to immunogenic properties (see below).

#### 2.1.3. External Factors Influencing EV Homeostasis

**Figure 3:**
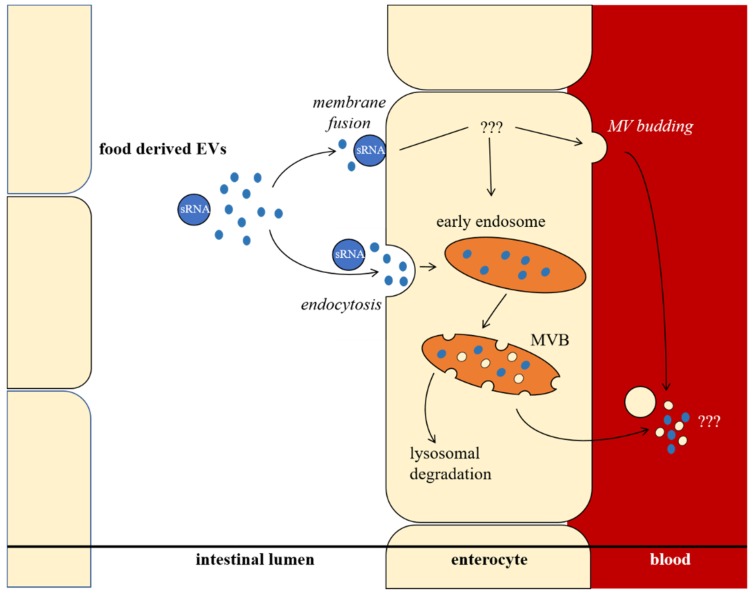
Intestinal resorption, processing, and release of extracellular vesicles (EVs). EVs can be absorbed by enterocytes via membrane fusion or endocytosis mechanisms. It remains ambiguous if food derived EVs can pass the intestinal barrier unprocessed, or if they induce changes inside the enterocyte, and therefore influence the cargo and release of enterocyte derived EVs. MVB, multivesicular body; sRNA, small noncoding RNA; MV, microvesicle.

In recent years, it has been demonstrated that plant secondary metabolites have effects on exosomal sRNA and EV levels in mammals. Recently, an overview has been given by Otsuka et al. [18]. More and more evidence suggests that food-derived EVs can influence human health. Since circulating sRNAs in body fluids can regulate metabolism and mRNA translation in the whole organism, it is conceivable that food-derived sRNAs, shuttled by EVs, spread their effects throughout the body [18,28]. It is still difficult to interpret how food-derived EVs or their cargo pass the intestinal barrier, since both membrane fusion and endocytosis are possible uptake mechanisms. How EV cargo is shuttled through or processed inside the enterocyte remains questionable, as schematically shown (Figure 3). Because the human diet usually consists of plants as a major component, plant EVs apparently have a comprehensive influence on human metabolism and on gut microbiota [78].

### 2.2. Plant EVs

“Exosome-like” EV populations have been found in distinct plants. EVs have been isolated from diverse organs like leaves, fruits, seeds, roots/rhizoma, pollen or semen [18,79,80,81,82,83,84], leading to the conclusion that EVs are in plants as ubiquitous as they are in animals.

Plant-derived EVs showed median diameters of ~400 nm for EVs from grapes, and ~250 nm for edible grapefruit and ginger nanoparticles. While both small (~100 nm) and large (~1000 nm) EVs were found in carrots [81,85]. Recent investigations of four *Citrus* L. species, by Pocsfalvi et al., have also shown small and large EV populations with significant differences in particle sizes and yields between the species, although they all belong to the same genus. The group divided between micro- and nano-vesicle fractions. Combined yields were determined as follows: grapefruit delivered 6.1 mg vesicle protein/mL fruit juice, orange 3.5 mg/mL, bitter orange 1.3 mg/mL, and lemon 0.8 mg/mL [86]. These data indicate that plants release EVs with species-specific sizing. The amounts of vesicles that are shed can probably be induced by external factors such as pathogen infection [27]. The influence of the size on particle properties cannot be overseen at this state of knowledge.

Observing plant EVs being shed from MVBs by electron microscopy [84] has proven that plants indeed shed genuine exosomes. Supporting evidence is delivered by the comparatively high similarity between the proteomes of EVs and MVBs/late endosomes [27]. Nonetheless, we have no information on plant-derived microvesicles and, so far, can only divide into small and large EVs or nano- and microvesicles, which is sufficient until more information is available. Furthermore, depending on the isolation technique, the vesicle´s origin can be ambiguous and EVs can be accompanied by intracellular vesicles.

The cell wall appears to be a barrier that cannot be overcome by EVs, but it only seems as such, because EVs can indeed be isolated from apoplastic fluids [27,79,87]. The hypotheses on how EVs pass cell walls, can assumedly be transferred from bacteria and fungi to plants. These are that EVs can be forced mechanically through the walls, because the membrane is not rigid, or that the wall thickness, integrity, or pore size is adapted to EV release. Another theory is that the EVs temporarily loosen the wall structure, since cell wall remodeling enzymes have been found in EV preparations [29,88,89,90,91,92]. Walker et al. demonstrated 60 nm to 80 nm liposomes penetrating the fungal cell wall, having a predicted pore size of approximately 5.8 nm. The group concluded that cell walls are less rigid than they are usually assumed to be. They are rather dynamic structures with flexible viscoelastic properties and permissiveness for vesicular structures in both directions, i.e., uptake and release [93].

#### 2.2.1. Plant EV Lipids

Lipidomic analysis of plant EVs has revealed a relatively unusual range of lipid compounds. Comparative TLC lipid profiling of EVs, isolated from grape, grapefruit, ginger, and carrot, has shown some characteristic bands for all species. The organ of origin also influenced the lipid profile [81]. EVs isolated from grapefruits (*Vitis vinifera* L.) were found to comprise 98% phospholipids (amongst them mainly phosphatidic acid (PA) with approximately 50%) and only 2% typical plant galactolipids. PA was shown to be mitogenic, as well as controlling membrane fusion and fission processes [80,85,94,95]. A study on grapefruit-derived EVs indicated an enrichment of phosphatidylethanolamine (45%) and phosphatidylcholine (28%), whereas PA was only slightly present with an amount of 2.5% [96]. Ginger EV lipids mainly consist of PA (~43%) and mono- and digalactosyldiacylglycerol (~46%) [72,95].

Membrane lipids are obviously determining EV stability. Differences in lipidomic profiles are possibly crucial for targeting certain recipients and might form the basis for interspecies and inter-kingdom communication. One can hypothesize that plants release EVs with different membrane composition to address different targets. This would, of course, not only affect lipids, but also cargo and proteome.

#### 2.2.2. Proteins of Plant EVs

The lack of protein markers for plant EV definition, determination, and isolation created increasing interest in their proteomic profile. Some proteins or protein families have been detected from independent groups in distinct plant species, such as patellins 1–3 [27,86], tetraspanin 8 [27,97], clathrin heavy chain [27,80,86,98], and heat shock proteins [27,80,86,96,98].

We have compiled frequently identified EV proteins (Table 1) which were isolated from distinct plant species. Future plant EV markers are probably among them.

#### 2.2.3. Applications of Plant EVs

Recently, upcoming evidence has shown that plant miRNAs and EVs are promising agents for therapeutic use. For example, grape EVs have shown beneficial effects on mouse intestine regeneration. Vesicles effectively induced proliferation of murine intestinal stem cells, when administered under pathological conditions. Mucosal epithelium regeneration was accelerated and the intestinal architecture rapidly restored throughout the entire length of the intestine [80]. Significantly, continuous oral administration protected mice from dextran sulfate sodium-induced colitis and treated mice lived twice as long as untreated mice [80,99]. Additionally, it has been shown that ginger-derived EVs are preferentially taken up by intestinal macrophages or monocytes, and therefore induce anti-inflammatory mediators. Dietary uptake of EVs from distinct fruits and vegetables probably provides greater beneficial effects for the maintenance of gut homeostasis than from single plant EVs [81]. Oral administration of ginger EVs protected mice from alcohol-induced liver injury, suggesting promising properties as a novel agent to prevent or even cure liver damage [100]. Nanosized vesicles isolated from *Citrus limon* (L.) Osbeck inhibited cancer cell growth in vitro and in vivo. It is remarkable that normal cells were not affected and angiogenesis was also inhibited [98]. This underlines the capability of inter-kingdom regulation through food-derived EVs and their potential as therapeutic vehicles. For this purpose, nanovectors were made of lipids, isolated from either grapefruit or ginger EVs. These nanovectors have shown several properties of major pharmaceutical interest, such as:

- no cytotoxic effects [71,72];
- higher uptake efficiency for the majority of investigated cells (even B and T cells), compared to liposomal formulation [71];
- no immune reaction detectable [71];
- no observable adverse effects [101];
- intranasal nanovector application delivered miR-17 to brain tumor cells within a short time in mice, whereas liposomes did not reach the brain [101];
- intravenous injection of nanovectors delivered miR-18a to liver macrophages and, consequently, promoted anti-tumor M1 macrophage induction [102];
- highly efficient cell internalization and cancer suppression of aptamer-doxorubicin loaded nanovectors [103]

Crucial advantages of EV packaged drugs have been revealed, such as increased stability, solubility, and bioavailability of hydrophobic agents, whereas no altering of the drug´s biological activity was recognized. Regarding biocompatibility and biodegradability, EVs offer sustainable materials [71], which can be harvested in large scale from plants and prospectively used for clinical applications in a highly safe and cost-efficient manner [95,102,104]. The findings on differing lipid profiles between plant EVs, together with the data on in vitro and in vivo effects lead us to the assumption that nanovectors could be specifically targeted by combining different plant EV derived lipids.

EVs from *Helianthus annuus* L. inhibited fungal spore germination, mycelial growth, and loss of vitality [91]. Furthermore, EVs appear to be enriched by plants in response to fungal infections [27,91,97] and antifungal Shogaol has been determined in ginger-derived EVs [100,105]. Combined, these data indicate that plants shed EVs into the apoplastic space as a kind of functional patrol unit, providing protection from fungal invaders. We can potentially use these antifungal properties of EVs in therapy of topical and systemic mycoses. An extended knowledge of EVs and the mechanisms of information transportation also provides a chance to control fungal plant pests, which can cost severe losses in cultivation of food plants, without toxicity for the environment, humans, and animals [106].

### 2.3. Fungal EVs

Although the first observations of fungal EVs were in the early 1970s, interest in them declined, to a 30-year slumber, and was reawaken in the beginning of the 21st century [6,107,108,109]. Fungal EV protein composition and morphology show similarities with mammalian exosomes [29]. Possibly, fungal EVs originate from membrane budding [110] or from cytosolic compartments [111]. EV formation in fungi has not been conclusively clarified, but it seems as if they release MVB-derived exosomes and membrane-derived microvesicles, similarly to animal cells [90,109,112,113].

As in plants, the passage of fungal EVs through the cell wall remains a mysterious, keeping scientists on tenterhooks, whereas hypotheses were adopted from one kingdom to another (see also Section 2.2 Plant EVs). Fungal EVs comprise RNA species, such as mRNA, tRNA, and sRNA, and protect them from RNase degradation [114], comparable with animal and plant EVs. Thus, fungal EVs are capable of cell-to-cell communication and inter-kingdom regulation [115,116]. It is assumed that EVs are strongly correlated to fungal virulence [90,108,117,118,119], since different mutants, with impaired EV secretion capability, showed less pathogenicity [120,121,122] and co-application of *Cryptococcus neoformans* (San Felice) Vuill. with additional EVs elevated fungal infectivity [123]. Unlike plant EVs, EVs from fungi can provoke an immune response, when administered in vivo. Thus, clinical use of fungal EVs offers potential vaccines against mycoses [90,118,119]. Fungal EVs have stimulated the release of the cytokines IL-4 and TNF-α in vivo [124], as well as nitric oxide, IL-10, IL-12 TNF-α, and fungicidal activity of macrophages in vitro [114,119,125].

#### Fungal EV Proteins and Lipids

Since fungal EV lipidomic and proteomic data have already been compiled [109,113], we want to point out some especially interesting facts, such as detected homologous proteins, which we estimated to be of particular interest in plant EVs. As such, clathrin heavy chain, heat shock proteins, syntaxins, and ESCRT complexes were identified in *Malassezia sympodialis* R. B. Simmons et E. Guého [126]. In addition to other proteins, heat shock proteins were found throughout many species, such as in *Candida albicans* (C. P. Robin) Berkhout [119], *Histoplasma capsulatum* Darling [117], *Paracoccidioides brasiliensis* (Splend.) F. P. Almeida [127], and *Saccharomyces cerevisiae* (Desm.) Meyen [128].

More remarkable are the results of lipid analysis, since phospholipids also appear in fungal EVs [113]. They were found in sterols, as well as in the neutral glycosphingolipid glucosylceramide in *C. albicans* [119], *H. capsulatum* [117], and *P. brasiliensis* [129]. The identification of glucosylceramide among EV lipids is of particular significance, due to the findings that *C. neoformans* loses its virulence, when lacking glucosylceramide synthase [130], and that it is essential for hyphal growth and spore germination for instance in *Aspergillus nidulans* (Eidam) G. Winter and *Fusarium graminearum* Schwabe [131,132]. Consequently, EVs appear to be substantially involved in fungal virulence and not merely the cargo but also the shell is decisive for EV functionality.

## 3. Prokaryotic EVs

### 3.1. Bacterial EVs

Sixty years ago, it was observed that cell-free supernatants of extracellular lipid-dense material from pathogenic *Vibrio cholera* Pacini culture contained parts of the bacterial outer membrane material with toxic effects on human cells [3]. Electron microscopy imaging revealed nanometer-sized droplets, which were budding from the bacterial cell wall [4]. These droplets are now known to be OMVs, a subcategory of EVs. In a stricter definition, only vesicles secreted from gram-negative bacteria are termed OMVs, whereas those from gram-positive bacteria are called microvesicles, however, the term OMV is now widely used.

All gram-negative and some gram-positive bacteria constitutively release OMVs, ranging in size from 20 nm to 250 nm in diameter, into the extracellular milieu [89,133]. The structure of OMVs is closely connected to the architecture of the (gram-negative) cell envelope. In general, OMVs are composed of an outer leaflet of lipopolysaccharide and an inner leaflet of phospholipid, decorated with membrane and surface proteins, and, as compared with human EVs, they additionally possess other structural features [134]. Indeed, OMVs have been shown to contain all sorts of cargos including cell wall components, peptidoglycans, outer membrane proteins, lipopolysaccharides, phospholipids, as well as soluble proteins (periplasmic, cytoplasmic) such as enzymes, nucleic acids (DNA, RNA) [135,136] and secondary metabolites [137]. OMVs are temperature and chemically stable entities [138]. Their composition, yield, and content can differ based on bacterial source and general growth conditions, such as nutrients, temperature, antibiotics, etc. [139]. Interestingly, even in the same culture under similar conditions, subpopulations of OMVs with different properties can be identified [139], a puzzling phenomenon that is still under discussion.

#### 3.1.1. Biogenesis of OMVs

Under natural conditions OMVs are produced rather passively as side products of cellular processes, but they may also be shed in response to stress, via explosive cell lysis [140], or in an active manner [141]. The exact mechanism which results in outer membrane budding remains unknown, and the following three models for OMV biogenesis are currently discussed:

- random budding during cell wall turnover;
- the stress response model;
- structural changes of lipopolysaccharides.

Cell wall turnover is a natural routine process in which the cell recycles cell wall components such as peptidoglycans. For this, the lipoprotein links between the outer membrane and the peptidoglycans have to be cut and rearranged leading to membrane protrusion and vesicles release from the cells surface into the extracellular space [142]. The stress response model is based on physical or chemical stress-induced malfunctioning membranes leading to accumulation of peptidoglycan fragments or misfolded proteins in the periplasm [143]. This leads to enhanced turgor pressure, membrane protuberances, and pinching-off of small membrane portions. This model supports the idea of OMV formation as a helpful waste mechanism to get rid of excess potentially harmful proteins [141]. Another model postulates that cations that cross-bridge the highly electronegative lipopolysaccharides, induce structural changes. Subsequent repulsion between lipopolysaccharides leads to local deformation and bacterial cell membrane shedding [144,145]. OMV production is a resource-depleting process, which probably would not be favored by evolution, if it was not for a purpose. Therefore, no models alone can comprehensively illuminate OMV production under natural conditions. Proteome analysis showed that OMV content could be enriched or depleted in comparison to the originating bacterial envelope fractions, indicating that OMV and outer membrane profiles are not necessarily identical [141] and that specific cargo can be actively sorted into OMVs. One model of active sorting is based on the discovery that the OMV production is not uniformly distributed along the outer membrane but concentrates on distinct areas or “hot spots” [133]. These hot spots were found to be locally enriched with specific proteins and lipids, while other vesiculation inhibiting proteins, such as lipoproteins for cell wall integrity, were reduced [144]. Some of the proteins involved in hyper- or hypovesiculation were identified by deleting genes potentially involved in OMV production, but direct evidence for active OMV biogenesis is still lacking. Most likely, different strains rely on diverse vesiculation triggers and mechanisms, which might have evolved separately [146]. The last step of vesiculation, the fission of formed vesicles, is an active, and thus energy dependent step, but as there is no energy source present in the periplasm, conformational changes in outer membrane proteins are thought to be involved [133].

#### 3.1.2. Function and Effects of OMVs

Many functions of OMVs are not yet elucidated. OMVs are generally considered to be distinct transport system but are more refined than just the secretion of free substances into the extracellular medium. Within OMVs, the natural cargo is protected and present at high concentrations being comparably unaffected by diffusion. OMVs can reach their target site both between neighboring bacteria and over long distances from the source bacteria [133]. OMVs affect their surroundings and play substantial roles in interspecies communication and cooperation including multicellular development, quorum sensing, and virulence factors [147,148]. OMVs also have defensive and offensive functions, such as lytic enzyme cargo to degrade prey bacteria for nutrition supply and for survival in complex environments [133,149]. They are thought to be “nucleation” centers to initiate and enhance biofilm formation [150] and also stabilize biofilms via network-like OMV chains. OMVs surrounding bacteria can protect from viral attack by mimicking cells and absorbing viruses [151]. OMV release can also serve as stress response to excrete misfolded, and hence potentially toxic proteins, as well as drugs including antibiotics [143,152,153].

#### 3.1.3. OMV Proteins

Due to the diversity of bacteria and the consequential plurality of OMVs, there are no universal markers known for the identification of OMVs [154]. Compared to membrane proteins from other sources, the outer membrane proteins of bacteria are not made of transmembrane α-helices but consist of antiparallel β-barrels [155]. In contrast to mammalian EVs, bacteria show larger diversity, interspecies differences in envelope composition, and architecture and bacterial processes, therefore, no single mechanism of OMV export is known, making it challenging to unravel basic mechanisms of vesicle transport. Some of the OMVs’ surface proteins are assigned to specific invasive abilities, such as internalization into the host cell membrane [156]. To mention a few, these proteins include invasins IpA, IpC, and IpaD, as well as outer membrane proteins from *Shigella flexneri* Castellani et Chalmers aimed at enhancing cellular uptake [157]. In *Escherichia coli* (Migula) Castellani et Chalmers, different outer membrane proteins (e.g., OmpA, AiL) were identified and shown to be required for pathogenesis via host receptor interactions [158]. Another invasive bacterial protein is ClyA, a pore forming toxin from *Salmonella* Lignières and *Escherichia* strains which has also been detected in OMVs [159]. A more comprehensive overview of outer membrane and bacterial proteins shed into OMVs can be found in recent reviews [133,156]. Once individually characterized, these OMV proteins may be used for vaccine developments or to enhance uptake of drugs into mammalian cells showing a potential therapeutic use [160].

### 3.2. Archaeal EVs

EVs from archaea can be assumed to occur in the same ubiquity as they do in the other empires of life. They have been described for *Sulfolobus* Brock et al. [12,161], *Ignicoccus* Huber et al. [162], *Thermococcus* Zillig [163], and *Halorubrum* McGenity et Grant [164] species and presumably more will follow.

On the one hand, the mechanisms of EV formation by archaea are ambiguous since, in *Sulfolobus* species, lipid and protein profiles between EVs and originating cell membranes revealed differences. Additionally, the finding of ESCRT III homologues indicated that EV release was a targeted process in *Sulfolobus* [161]. Moreover, the recognition of antimicrobial proteins in archaeal EVs indicates that they play a role as defensive agents [165]. On the other hand, EVs isolated from *Thermococcus* showed high similarities of proteins and lipids between originating membrane and EVs themselves. Combined with electron microscopic images, this perception suggested *Thermococcus* EVs result from membrane budding. This process is not assumed to be less specific, since minor differences, for example, in lipid composition were detected [163]. These two mechanisms of EV release are not contradictive, as animal EVs are known to originate either from MVBs or from membrane budding. Whether there are parallels to EV formation machinery in eukaryote [166] or there are archaea species capable of diverse EV release mechanisms, is still puzzling.

While EV lipids from other empires comprise phospholipids (besides other components), archaeal EV envelopes consist of diglycerol di(tri)alkyl tetraethers, similar to membranes of archae [161,163]. *Thermococcus* EVs have been shown to carry DNA, prevent DNA thermodenaturation [167], and enable the transfer of DNA and, presumably, other molecules [163,168].

Archaeal EV proteins have not yet been the focus of research and, so far, just a few have been identified. Among them, peptide binding receptors were found to be prominently abundant in *Thermococcus* and *Sulfolobus* EVs [163,168]. ATP binding cassette (ABC) transporters have also been found in *Thermococcus* EVs [163,168], which is remarkable because these proteins were commonly detected in EVs from eukaryote [27,86,98]. Due to the severe differences in the proteomic profiles of EVs from different species even within the same genus [163,168] and the extent of limited data, it remains to be determined if universal marker proteins for archaeal EVs exist.

## 4. Inter-Individual, Interspecies, and Inter-Kingdom Regulation

After the observation that EV-mediated information transfer is not limited to one organism, species or kingdoms, the one central question of EV research became, “Why can EVs overcome kingdom boundaries?” Investigations on EV-mediated regulation processes, from mother–infant to host–pathogen interaction, might elucidate this query (Table 2).

In addition to mitogenic lipids and signaling proteins, sRNAs are considered to be crucial regulatory elements in EV-mediated (inter-kingdom) communication [99]. They are able to manipulate various biological processes, such as cell growth, differentiation, development, metabolism, and apoptosis [19,20]. Stability and absorption of sRNA are obviously critical aspects of bioavailability for recipient organisms or cells. In contrast to traditional persuasions on the stability of extracellular RNA, a few studies have shown surprisingly high pH-, temperature-, and RNase-resistances for sRNA in mammalian body fluids [26,190,191,192,193,194], as well as for plant sRNAs [21,80,96,195,196]. The vesicular envelope of EVs is thought to be decisive for the enhanced sRNA stability. This assumption is strongly underlined by the fact that severe losses of sRNA are detectable after pasteurization and homogenization or after ultrasonic exosome depletion of bovine milk [28,175,197]. Furthermore, the envelope also provides a vehicle for cellular uptake of the cargo, not only in the intestine [28,175,176,192,198,199,200].

Since EVs have been found in the milk of distinct mammals, such as pork, cow, or human, increasing numbers of inter-individual and interspecies regulation processes are being assumed highly probable [28,192,201,202,203,204]. Moreover, increased serum levels of bovine milk specific sRNA were detected in humans after consumption of cow´s milk [175]. Until today, we are lacking reliable studies on physiological or pathological effects of ingested EVs on humans, while a broad range of such effects is conceivable. This assumption is supported by investigations that have shown that a breastfed infant profits from ingested milk-derived sRNAs by elevated T-cell levels and enhanced differentiation of B cells [20,28,192,201].

Although there has been previous evidence for inter-kingdom regulation mediated by sRNAs [205,206,207,208], the study by Zhang et al., 2012 was somehow paradigm shifting. Their finding, that the dietary uptake of a particular plant-derived micro RNA can measurably affect the metabolism of a mammal [189], quickly ignited increased interest in this field.

Probably, fungal cells send EVs in order to downregulate host immune response. Observations in both human–fungus and plant–fungus interactions suggest fungal virulence to be strongly enhanced by inter-kingdom RNA interference, enabled by sRNA containing EVs [124,172,179,181,209]. Conversely, plants send sRNA to silence fungal virulence genes, which has recently also been related to EVs [27,91,97,106,210,211].

In the area of difficult-to-treat infections, OMVs play a major role in drug resistance because they transfer resistance genes (DNA) between bacteria, even of different origin [148]. Many OMVs from pathogenic bacteria were found to have surface proteins, which can readily interact with mammalian host cells. These interaction mechanisms make OMVs a pivotal element of trans-kingdom and host-cell communication by letting them interact in a highly specific manner [212]. OMVs have been shown to carry PAMPs, including lipopolysaccharides, and can transfer other virulence associated factors [213]. These factors can trigger strong immune responses in host cells, while OMVs act as immunomodulators, for example, by leading to expression of receptors on macrophages to specifically recognize the pathogen [214]. As OMVs can help pathogenic bacteria to persist attack by the mammalian immune system, they strongly contribute to the cause of infectious disease [174,182]. Prokaryotic pathogens such as *Bacillus anthracis* Cohn [183], *Helicobacter pylori* (Marshall) Goodwin [184], *Neisseria gonorrhoeae* (Zopf) Trevisan [185], *Pseudomonas aeruginosa* (Schroeter) Migula [186], and *Streptococcus pneumoniae* (Klein) Chester [187], as well as eukarytotic pathogens such as *Leishmania spp*. Ross [215], *Plasmodium spp*. Marchiafava et Celli [216], and *Trichomonas vaginalis* Donné [217] similarly send EVs to increase their contagiousness [188,218,219,220]. This phenomenon is not limited to unicellular organisms, since helminths also modulate host immunity, as *Heligosomoides polygyrus* Dujardin [26] and *Dicrocoelium dendriticum* Rudolphi [177].

**Figure 4:**
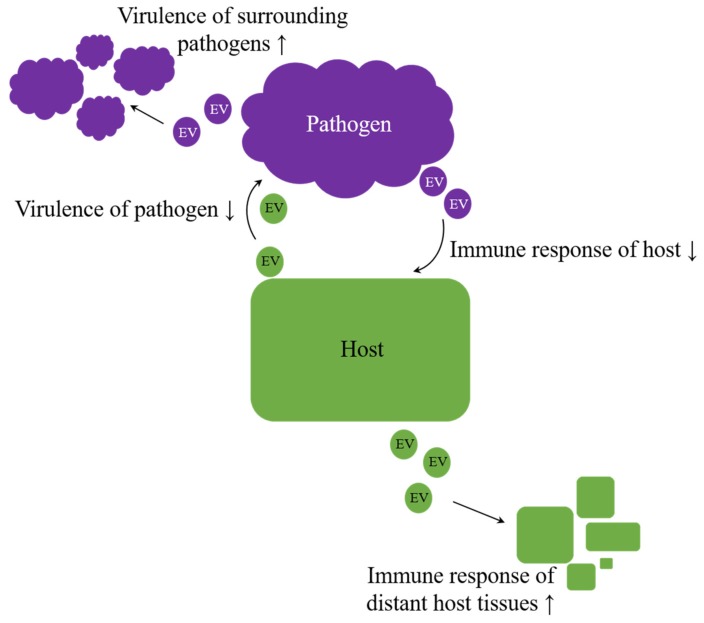
Arms race in host–pathogen interaction. Irrespective of kingdom boundaries, the genuine role of extracellular vesicles (EVs) appears to be bilateral. On the one hand, they were proven to have protective properties, but, on the other hand, they also appear to contribute to the achievement of inherent aims, like enhancing virulence on pathogens side or improving host´s immunity.

Overall, EVs appear to be potent agents in regulation processes, crossing not only the borders of species but rather of kingdoms or even empires. Therefore, they enhance an arms race in host–pathogen interaction [106,180]. But do exosomes also facilitate intercellular communication beyond the animal kingdom? Especially host–pathogen interactions imply the possibility of host-host and pathogen-pathogen signaling, intended to improve the chance of survival on each side (Figure 4). A better understanding of host-pathogen interactions can elucidate unknown mechanisms, and therefore future targets, improving therapies of infectious diseases.

## 5. Conclusions

Because shedding of EVs has been found to be ubiquitous throughout all empires of life, it appears to be evolutionarily advantageous. The abundance of homologous proteins in distinct kingdoms clearly indicates that the release of membranous vesicles is evolutionary highly conserved. Independently from their origin, EVs can be loaded with a wide range of drugs, including chemotherapeutic compounds, DNA expression vectors, sRNA, and proteins such as antibodies, and have been shown in vivo to deliver their cargo and to protect the therapeutic agent from degradation [71,102,221,222]. Because the application of EVs can either increase or decrease the in vitro viability of cells, the bioactive cargo seems to be responsible for the triggered effects. Lacking cytotoxic effects, edible plant-derived EV lipids are interesting for the development of nanovectors regarding drug delivery. But since our knowledge of the comparability of EVs from different kingdoms is limited, comprehensive EV research regarding multiple organisms offers a better understanding of the entire field.

The EV shell is generally assumed to be crucial for the stability of sRNA or rather the complete cargo. In animals, EVs are widely thought to facilitate intercellular communication, but we can only speculate about the “genuine intention” of EV release. There is evidence that EV-mediated inter-kingdom regulation is more than a random event. It seems to be more likely that cells release EVs in order to control (remotely) or influence their environment. Possibly, plants are using EVs as a defense strategy against invading fungi, while fungi for their part enhance their own virulence. Therefore, the composition of membrane lipids and proteins seems to be crucial for addressing the intended target cell, tissue, or organism. EVs consist of a complex and mutually well-coordinated mixture of biomolecules. They can be assumed to be Janus-faced natural products and it is on us to use this instrument in a responsible manner. Currently, we are at the beginning of a developing field and a comprehensive view on the issue could help overseeing complex linkage. Around 20 years ago, a couple of researchers realized EVs to be more than tiny garbage bags. They recognized their broad capability and kept going deeper into the unknown. As a result, we find ourselves today with very detailed knowledge on human exosomes. Unfortunately, this knowledge cannot be transferred one-for-one from animal EVs to other kingdoms, but those other fields can profit from well-established methods. This will ease the way towards unpredictable findings. Thus, now, we need the same pioneering spirit and courageousness to create a more general point of view, in order to fully exploit the potential of the cross-linking vehicles we try to decrypt.

## Figures and Tables

**Table 1:** Proteins and protein families found repeatedly in plant EVs.

Protein Family	Protein	References
Actins	Actin	[82,98]
	Actin-1	[82]
	Actin-7	[27,86]
	Actin-101	[86]
Annexins	Annexin	[91]
	Annexin A7/11	[98]
	Annexin D1, D2, D2-like	[27,86]
Aquaporins	PIP	[98]
	PIP 1-2	[27]
	PIP 1-3, 2-2, 2-4	[80]
	PIP 1-5, 2-1, 2-3, 2-5, 2-6	[27,86]
	TIP	[98]
	TIP1-1	[86]
Clathrin heavy chain	Clathrin heavy chain *	[91,98]
	Clathrin heavy chain 1 *, 2 *	[27,86]
Coatomer	Coatomer subunits alpha, beta, gamma, delta, epsilon and isoforms	[86,98]
Heat shock proteins	Heat shock protein 90 *	[86,91,96,98]
	Heat shock 70 kDa protein 3, 5- like, 14-like	[27,86]
	Heat shock protein 70 *	[82,91,98]
	Heat shock cognate 70 kDa protein 1 *	[27,80]
Patellins	Patellin 1 *, 2 *	[27]
	Patellin 3 *, 3- like *	[86]
Ras related proteins	RABA2a, 2b RABB1c Rab 7	[27,86]
	Rab-2A, 5C, 6A, 7A, 8A, 11A, 18	[98]
Syntaxins	(GFP-) Penetration 1 * Synaptotagmin A	[27]
	Synaptobrevin homolog Syntaxin of plants 5 Novel plant SNARE	[98]
	Vesicle transport v-SNARE 11, 13	[27,86]
others	CHMPs 1, 4, 6 ESCRT-I complex subunits TSG101, VPS28, VPS37 Vesicle-associated membrane proteins 7, 72	[98]
	Tetraspanin 8 *, 9, 18	[27,97]
	Vesicle-associated protein 4-1	[27]

CHMP, charged multivesicular body proteins; ESCRT, endosomal sorting complexes required for transport; PIP, plasma membrane intrinsic proteins; (v-) SNARE, (vesicle) soluble *N*-ethylmaleimide-sensitive-factor attachment receptor; TIP, tonoplast intrinsic proteins; and TSG, tumor susceptibility gene, * Proteins of particular interest.

**Table 2:** EV mediated regulation processes.

EV Mediated Regulations	Examples		References
Inter-individual regulation	mother ↔ foetus		[169]
	mother → infant regulation		[28,170,171]
	elevated fungal virulence		[172,173]
	elevated bacterial virulence/drug resistance within the same species		[148,153,156,174]
Interspecies regulation	dietary uptake, e.g., bovine milk → other mammals		[175,176]
	pathogen-host interactions, e.g., helminth ↔ animal host		[26,177]
	elevated bacterial virulence/drug resistance beyond species boundaries		[148,153,156,174,178]
	archaeal antimicrobial proteins inhibit growth of other archaea		[12,165]
	archaeal DNA tranfer		[163,168]
Inter-kingdom regulation	pathogen–host interactions:	plant ↔ fungus	[27,91,97,179,180,181]
		animal ↔ fungus	[119,124,172]
		bacteria ↔ animal	[156,174,182,183,184,185,186,187,188]
	dietary uptake, e.g., rice → mammal		[18,189]
	archaeal antimicrobial proteins inhibit bacterial growth		[12,165]

## Abbreviations

ABC	ATP binding cassette
CHMP	charged multivesicular body protein
ESCRT	endosomal sorting complex required for transport
EV	extracellular vesicle
ISEV	International Society for Extracellular Vesicles
MISEV	minimal information for studies of extracellular vesicles
MSC	mesenchymal stem cell
MVB	multivesicular body
OMV	outer membrane vesicle
PA	phosphatidic acid
PAMP	pathogen-associated molecular pattern
RNAi	RNA interference
SNARE	soluble *N*-ethylmaleimide-sensitive-factor attachment receptor
sRNA	small noncoding RNA
